# Supplementary material for: CHML promotes liver cancer metastasis by facilitating Rab14 recycle
Source: Nat Commun. 2019 Jun 7;10:2510. doi: 10.1038/s41467-019-10364-0 (PMC6555802; doi:10.1038/s41467-019-10364-0)
Supplement: Supplementary file 1 — Supplementary Information [file 41467_2019_10364_MOESM1_ESM.pdf]

CHML promotes liver cancer metastasis by facilitating Rab14 recycle

Chen *et al.*

Supplemental materials: 8 figures; 1 table.

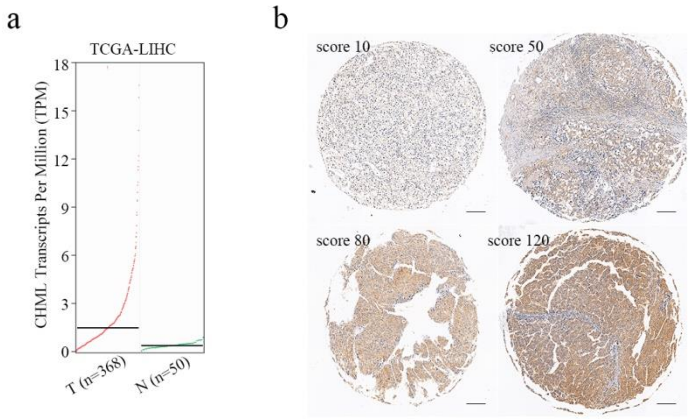

Supplementary Figure 1. CHML is upregulated in HCC. (a) Dot plot analysis of CHML expression in TCGA liver cancer dataset. (b) Representative images of tissue microarray stained with CHML and scored by standard procedure. Scale bar, 100μm. All data are represented as mean ± SEM.

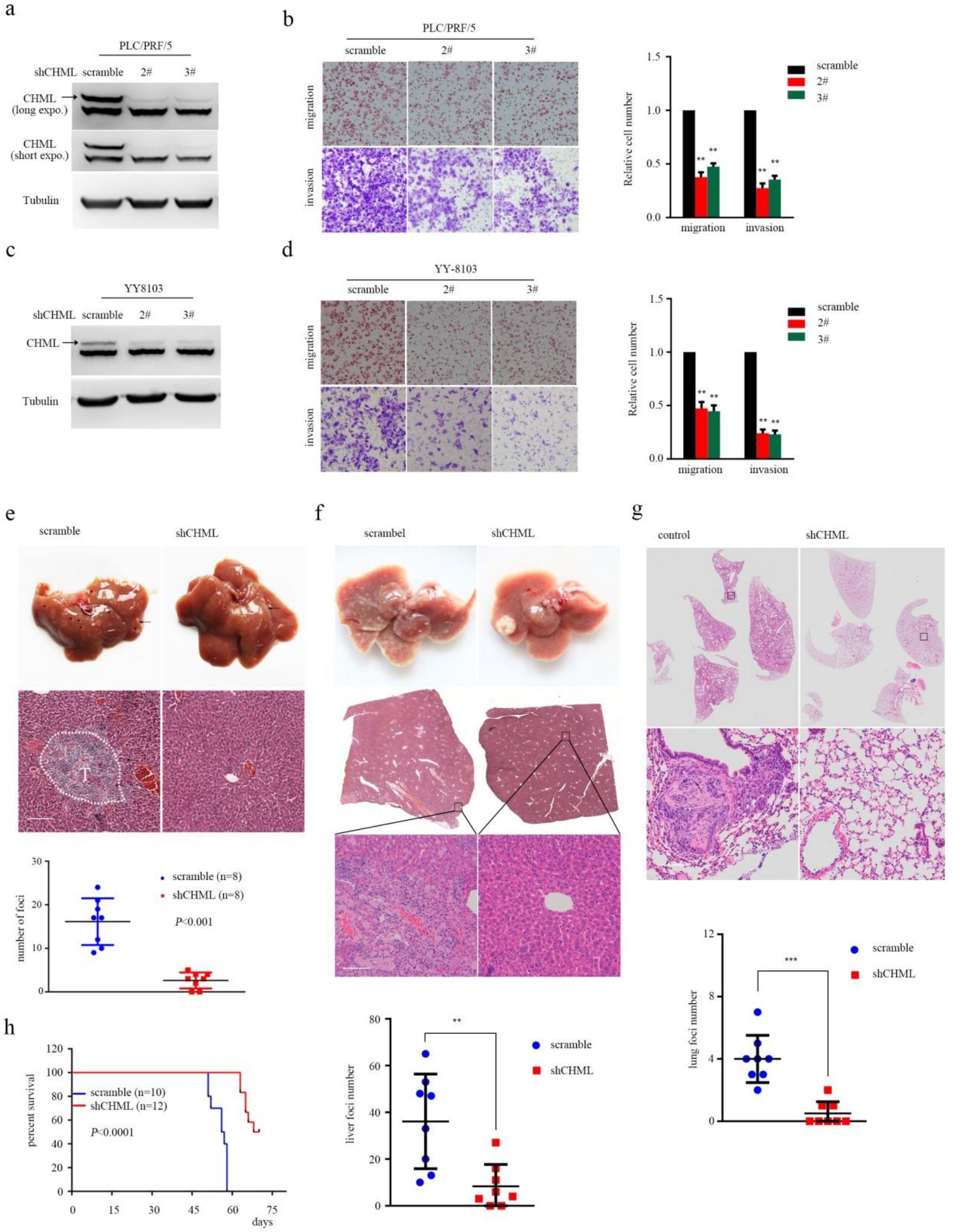

Supplementary Figure 2. Knockdown of CHML in HCC cells decreased cellular migratory and invasive ability both in vitro and in vivo. (a, c) Western blot analysis of the knockdown efficiency of CHML in HCC cell line PLC/PRF/5 (a) and YY8103 (c). (b, d) Boyden chamber and invasion assay are conducted to detect the migration and invasion abilities of control cells and CHML-knockdown PLC/PRF/5 cells (b) and YY8103 cells (d). Each representative image is shown. Data are shown as the mean ± s.e.m., ***P*<0.01 by two-tailed unpaired Student's *t*-test. (e, f) Livers of intrahepatic metastasis model with YY cells (e) or LM3 cells (f) are photographed, fixed and stained with haematoxylin and eosin (HE). Black arrowhead indicates intra-hepatic metastasis, arrow indicates primary injection site. Tumor region is indicated by white circle. Scale bar, 100

μm. The number of metastatic lesions in each specimen is counted and plotted. **P<0.01 by two-tailed unpaired Student's t-test. (g) HE staining of lungs from both control and shCHML groups resected from tail vein injection metastasis mouse model of LM3 cells. Scale bar, 100 μm. ***P<0.001 by two-tailed unpaired Student's t-test. (h) Kaplan-Meier survival analysis of mice injected with control cells and shCHML cells. All data are represented as mean ±SEM.

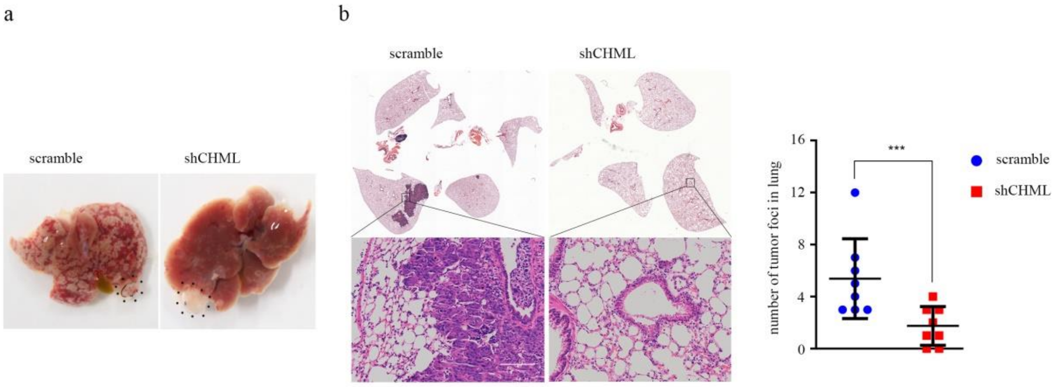

Supplementary Figure 3. Knockdown of CHML in LM3 decreased its migration ability in orthotopic xenograft model. (a) Representative photos of livers resected from nude mouse 45 days post tumor implantation. Dotted circle indicates the implanted tumor tissues. (b) HE staining of lungs from the orthotopic xenograft mice. Scale bar, 100μm. Statistics were shown on the right. ***P<0.001 by two-tailed unpaired Student's t-test. All data are represented as mean ±SEM.

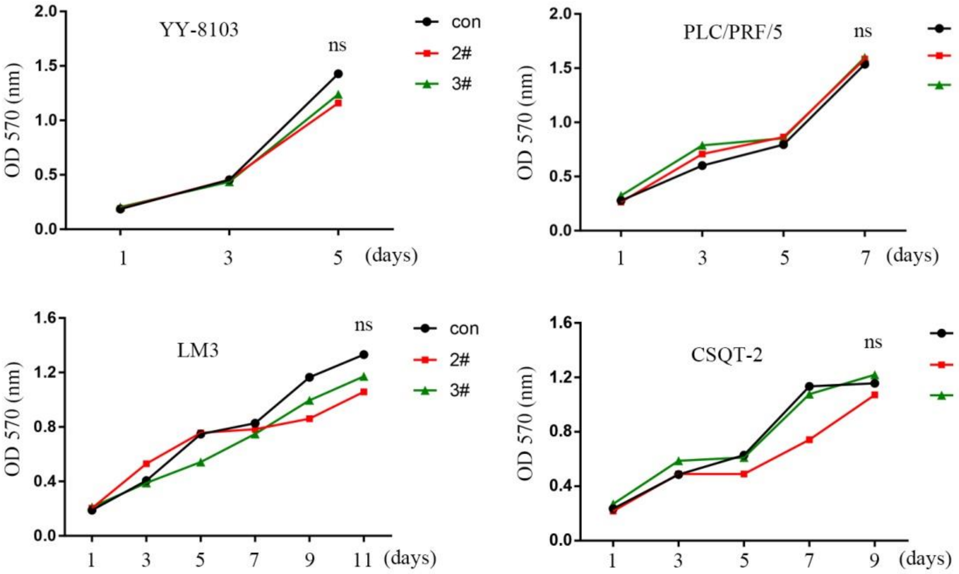

Supplementary Figure 4. CHML KD did not influence HCC cell growth. MTT assay was conducted to determine the proliferative ability of CHML KD HCC cells. ns, no significance.

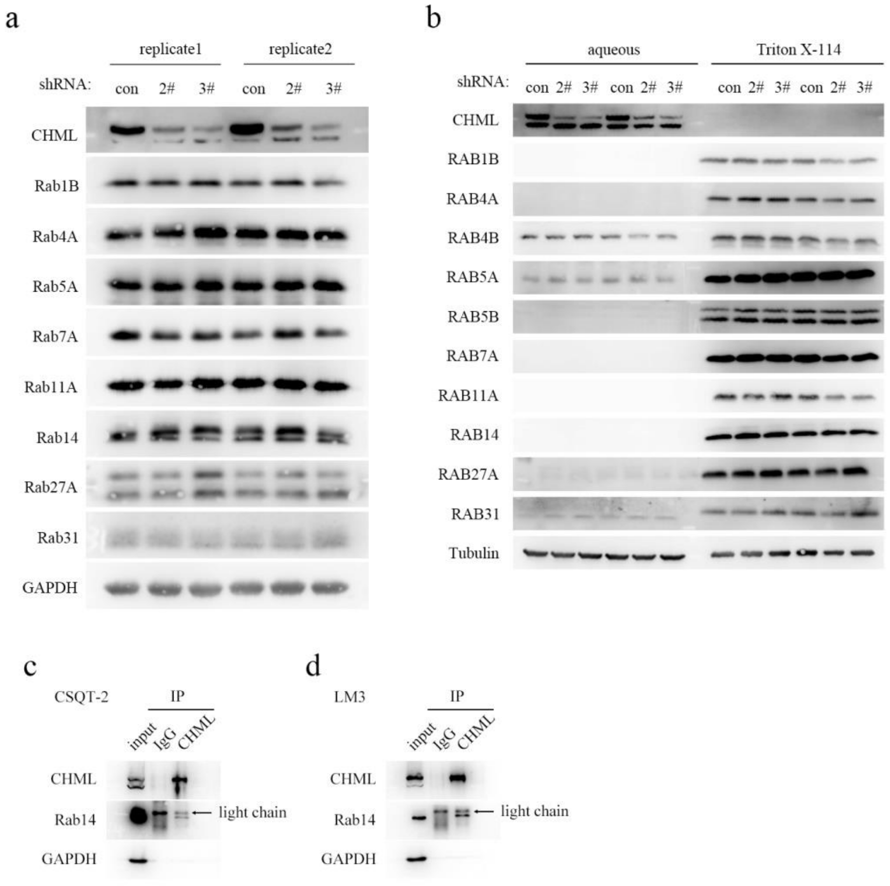

Supplementary Figure 5. CHML knockdown (KD) doesn't affect total and

geranylgeranylated Rab proteins. (a) Western Blot (WB) analysis of total Rab proteins. Two independent replicates were assayed. (b) WB analysis of geranylgeranylated Rabs (Triton X-114) and unprocessed Rabs (aqueous). (c, d) Endogenous interaction between CHML and Rab14 in CSQT-2 cells (c) and LM3 cells (d). Western blot result shows the IP result. Arrows indicates light chains.

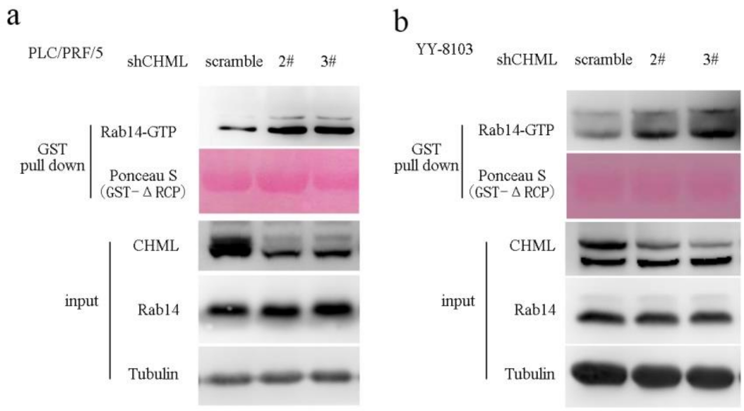

Supplementary Figure 6. CHML KD accumulates RAB14-GTP in cells. (a, b) Western blot analysis of Rab14-GTP in CHML KD PLC/PRF/5 (a) and YY-8103 cells (b).

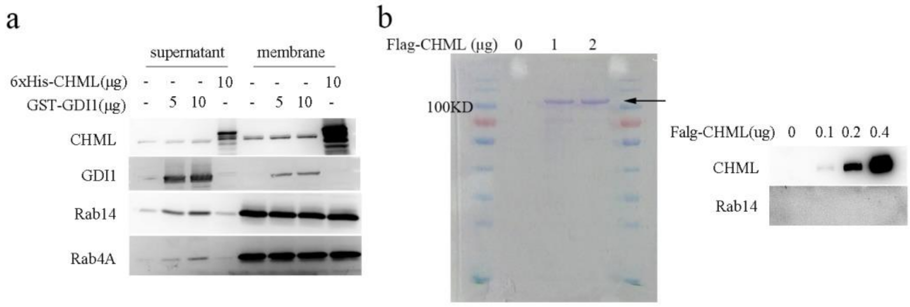

Supplementary Figure 7. CHML cannot extract Rab14 from membrane. (a) WB analysis of extraction assay. Membrane fraction were incubated with indicated proteins, and Rab14, Rab4A were resolved by WB. (b) Coomassie blue staining of Flag-CHML protein purified from 293T cells (right). WB analysis of Flag-CHML protein purified from 293T cells (left). Arrow indicated CHML.

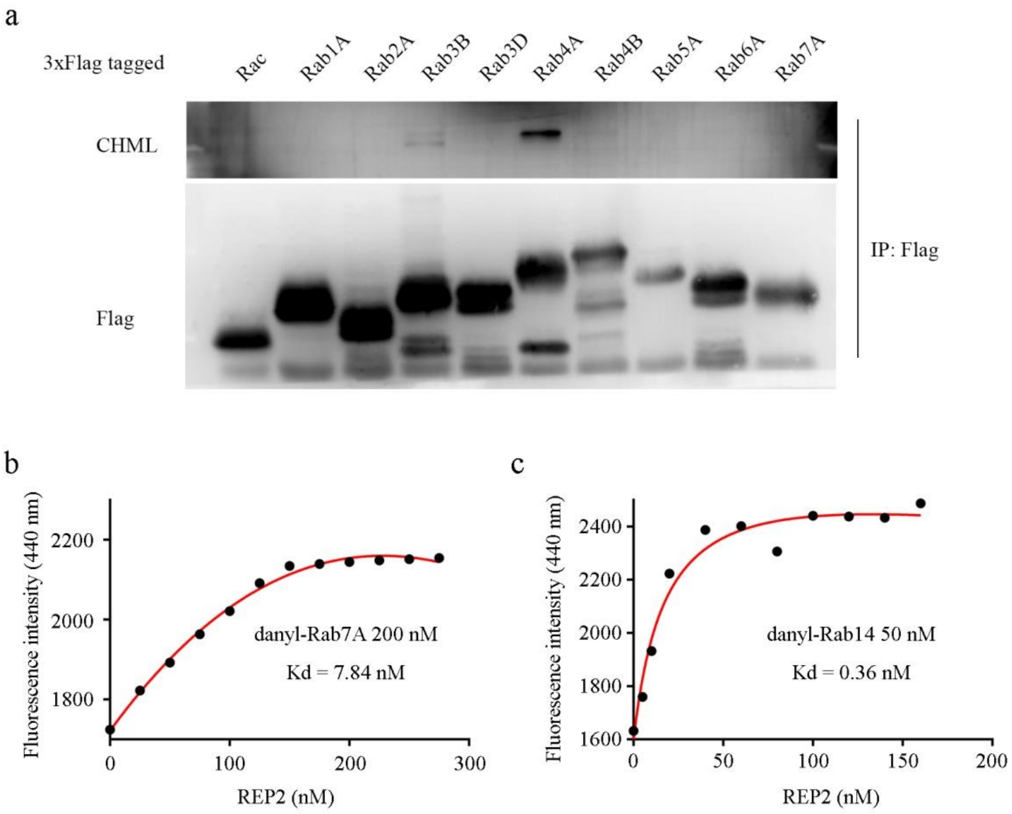

Supplementary Figure 8. Dissociation constant of REP2 binding to Rab7A/Rab14 determined by fluorescence titration assay. (a) WB result showing different Rab proteins binding to CHML in 293T cells. (b) Dot plot showing REP2 binding to Rab7A. (c) Dot plot showing REP2 binding to Rab14.

Supplementary Table 1. Primers used in this study.

Primer name (F: Forword primer; R: Reverse primer)	Sequence (5’------------>3’)
Real time-CHML-F	TCCCCAGGGTTTCTGTAGGA
Real time-CHML-R	TGGCTCTGCTGGAGGAACTA
Real time-actin-F	GATCATT GCTCCTCCTGAGC
Real time-actin-R	ACTCCTGCTTGCTGATCCAC
ORF-CHML-F	GGGGTACCCATGGCGGATACTCTCCCTTCGGAGT
ORF-CHML-R	GCTCTAGATTATTCAGAGGACTCCTCTAGGTTT
pGEX4T1-GDI1-F	CGGGATCCATGGACGAGGAATACGATGTGATCG
pGEX4T1-GDI1-R	CCGCTCGAGTCACTGCTCAGCTTCTCCAAAGACG
ORF-Rab4A-F	CGGGATCCATGTCGCAGACGGCCATGTCCGAAA
ORF-Rab4A-R	CCGCTCGAGCTAACAACCACACTCCTGAGCGTTC
ORF-Rab7A-F	CGGGATCCATGACCTCTAGGAAGAAAGTGTTGC
ORF-Rab7A-R	CCGCTCGAGTCAGCAACTGCAGCTTTCTGCCGAG
ORF-Rab14-F	CGGGATCCATGGCAACTGCACCATACAACTACT
ORF-Rab14-R	CCGCTCGAGCTAGCAGCCACAGCCTTCTCTCTGG
Rab14S25N-F	GGACATGGGAGTAGGAAAAAATTGCTTGCTTCATC
Rab14S25N-R	ATGAAGCAAGCAATTTTTTCCTACTCCCATGTCC
Rab14Q70L-F	AGATTTGGGATACGGCAGGACTGGAGCG
Rab14Q70L-R	CCGTGTAACAGCCCTAAATCGCTCCAGTCCTG
CHML-1-100aa-F	CGGGATCCATGGCGGACAATCTTCCCACAGAGT
CHML-1-100aa-R	CCGCTCGAGCTATTCTGTGTGTTGAATAGTTTCA
CHML-101-555aa-F	CGGGATCCGCTTTTTGCTACGCCAGTCAGG
CHML-101-555aa-R	CCGCTCGAGTTAATTAAAATAAAGAGCCCACAAGAG
CHML-556-656aa-F	CGGGATCCATGAGAGATTCCTCGGGAATCAGCA
CHML-556-656aa-R	CCGCTCGAGCTAATTTTGAAGGTGCTTCTCTGGG
RCP526-649aa-F	CGGGATCCCCAATTTCCTCTCCGAGGGCTCCCC
RCP526-649aa-R	CCGCTCGAGTTACATCTTTCCTGCTTTTTTGCCA
shCHML-2-F	gatccGCTTTCAGGCAGTGTTCATTTTCAAGAGAAATGAACACTG CCTGAAAGTTTTTTCTCGAGg
shCHML-2-R	aattcCTCGAGAAAAAACTTTCAGGCAGTGTTCATTTCTCTTGAA AATGAACACTGCCTGAAAGCg
shCHML-3-F	gatccGTAGGATGTGTGCAGTTTTTTCAAGAGAAAAACTGCACAC ATCCTACTTTTTTCTCGAGg
shCHML-3-R	aattcCTCGAGAAAAAAGTAGGATGTGTGCAGTTTTTCTCTTGAA AAAACTGCACACATCCTACg
CRISPR-Rab14-1-F	CACCGAAGATGTAAGAGTAGTTGTA
CRISPR-Rab14-1-R	AAACTACAACTACTCTTACATCTTC
CRISPR-Rab14-2-F	CACCGGTTACACGGAGCTACTACAG
CRISPR-Rab14-2-R	AAACCTGTAGTAGCTCCGTGTAACC
