# Supplementary material for: CHML promotes liver cancer metastasis by facilitating Rab14 recycle
Source: Nat Commun. 2019 Jun 7;10:2510. doi: 10.1038/s41467-019-10364-0 (PMC6555802; doi:10.1038/s41467-019-10364-0)
Supplement: Supplementary file 3 — Description of Additional Supplementary Files [file 41467_2019_10364_MOESM3_ESM.docx]

**Description of Additional Supplementary Files**

**File Name: Supplementary Data 1**

**Description:** Proteins enriched in IgG and Rab14 endosomes.

**File Name: Supplementary Data 2**

**Description:** Proteins in Rab14-positive endosome.

**File Name: Supplementary Data 3**

**Description:** Metastasis-related proteins in Rab14-positive endosome.
